# Development of a national point-of-care ultrasound training course for physicians in Japan: A 3-year evaluation

**DOI:** 10.12688/mep.19679.1

**Published:** 2023-10-25

**Authors:** Toru Yamada, Taro Minami, Yuka Kitano, Shunpei Yoshino, Suguru Mabuchi, Nilam J. Soni

**Affiliations:** 1 General Internal Medicine, Tokyo Bay Urayasu Ichikawa Medical Center, Urayasu, Chiba, 279-0001, Japan; 2 General Medicine, Graduate School of Medical and Dental Sciences, Tokyo Medical and Dental University, Bunkyo-ku, Tokyo, 113-8510, Japan; 3 Medicine, Division of Pulmonary, Critical Care, and Sleep Medicine, The Warren Alpert Medical School of Brown University, Providence, Rhode Island, 02903, USA; 4 Medicine, Division of Pulmonary, Critical Care, and Sleep Medicine, Care New England Health System, Providence, Rhode Island, USA; 5 Emergency and Critical Care Medicine, St. Marianna University, School of Medicine, Kawasaki, Kanagawa, 216-8511, Japan; 6 General Internal Medicine, Iizuka Byoin, Iizuka, Fukuoka Prefecture, 135-0041, Japan; 7 Medicine, Division of Hospital Medicine, The University of Texas Health Science Center at San Antonio, San Antonio, Texas, 78229, USA

**Keywords:** point-of-care ultrasound, training course, standardized training, Japan

## Abstract

**Purpose**: Point-of-care ultrasound (POCUS) allows bedside clinicians to acquire, interpret, and integrate ultrasound images into patient care. Although the availability of POCUS training courses has increased, the educational effectiveness of these courses is unclear.

**Methods**: From 2017 to 2019, we investigated the educational effectiveness of a standardized 2-day hands-on POCUS training course and changes in pre- and post-course exam scores in relationship to participants’ (n = 571) clinical rank, years of POCUS experience, and frequency of POCUS use in clinical practice.

**Results**: The mean pre- and post-course examination scores were 67.2 (standard deviation [SD] 12.3) and 79.7 (SD 9.7), respectively. Higher pre-course examination scores were associated with higher clinical rank, more years of POCUS experience, and more frequent POCUS use (p < 0.05). All participants showed significant changes in pre- to post-course exam scores. Though pre-course scores differed by clinical rank, POCUS experience, and frequency of POCUS use, differences in post-course scores according to participant baseline differences were non-significant.

**Conclusion**: A standardized hands-on POCUS training course is effective for improving POCUS knowledge regardless of baseline differences in clinical rank, POCUS experience, or frequency of POCUS use. Future studies shall evaluate changes in POCUS use in clinical practice after POCUS training.

## Introduction

Point-of-care ultrasound (POCUS) is a focused examination where ultrasound images are acquired, interpreted, and integrated into patient care by a clinician at the bedside. POCUS emerged in the 1990s as a useful bedside tool to guide clinical management. Since then, its use has expanded across nearly all specialties and includes a broad range of applications. In recent years, access to portable ultrasound machines has improved substantially as handheld, pocket-sized devices with high performance and lower cost have become available ( Soni *et al.*, 2020).

Despite the growing body of evidence supporting POCUS use and increasing availability of POCUS devices, lack of training continues to be the primary barrier to POCUS use among physicians in-practice ( Nathanson *et al.*, 2023; Wong *et al.*, 2020). Societies including the Society of Hospital Medicine, American Collage of Chest Physicians, and American College of Physicians have published guidelines and developed POCUS training programs for physicians in-practice ( American College of Physicians, 2023; Greenstein *et al.*, 2017; Society of Hospital Medicine, 2023; Soni *et al.*, 2019). Hands-on POCUS training courses have also gradually begun to spread in Asia ( Yamada *et al.*, 2018; Yamada, 2020). POCUS training standards have been developed for some specialties, but there is neither a broadly accepted standardized curriculum nor a national post-graduate certification accepted across all specialties ( Singh *et al.*, 2022; Soni *et al.*, 2020).

Most POCUS training courses are attended by clinicians of different specialties and with varying backgrounds with respect to clinical rank and prior POCUS experience ( Yamada *et al.*, 2018). There are few POCUS training courses that have objectively evaluated the effectiveness of the course ( Singh *et al.*, 2022), and it is not clear whether these courses are effective in improving POCUS skills of participants from different backgrounds. The purpose of this study was to examine the effectiveness of a standardized POCUS training course on POCUS skills of medical students, residents, and faculty physicians with differences in baseline clinical rank, years of POCUS experience, and frequency of POCUS use.

## Methods

### Design

We held eleven 2-day, hands-on POCUS training courses in Japan from 2017 to 2019. The course was held three times in 2017 (in Chiba, Gifu, and Aichi prefectures) and four times in 2018 and 2019 (in Chiba, Aichi, Gifu, Kanagawa in 2018 and in Fukuoka, Aichi, Saitama, Tokyo in 2019). The course was modeled after the Society of Hospital Medicine and American College of Chest Physicians POCUS courses and was accredited by the Japanese Society of Hospital General Medicine and the Japanese Association for Acute Medicine ( Yamada *et al.*, 2018).

**Figure 1.**
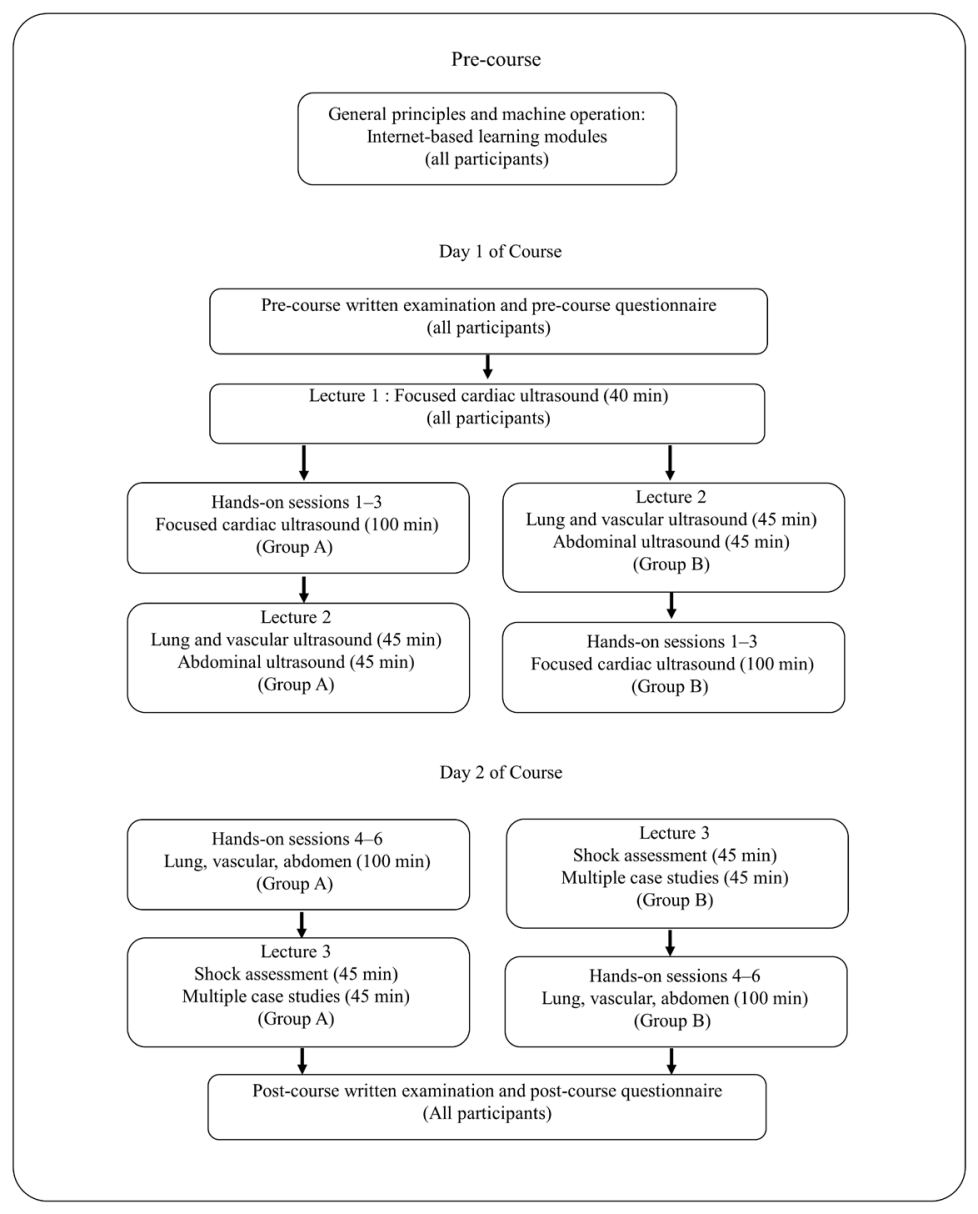
Point-of-care ultrasound training course schedule.

All participants completed internet-based modules on general principles of ultrasound and operation of an ultrasound machine before attending the hands-on course. The course started with a pre-course exam, followed by live lectures and hands-on sessions covering focused cardiac ultrasound (FOCUS); lung, vascular, and abdominal ultrasound; shock assessment, and multiple case studies, followed by a post-course exam ( Figure 1). The hands-on sessions involved 3 participants and 1 instructor (3:1 ratio) per skill station with a live model. All instructors passed a qualifying instructor examination, and an instructor handout was used to standardize instructional content and terminology. During the hands-on sessions, a course director circulated between stations to ensure consistency and quality of instructional content. The content of the hands-on sessions is shown in Table 1.

**Table 1.** Learning content of hands-on sessions.

Session no.	Domain	Learning content
1–3	FOCUS	Acquisition of PLAX, PSAX, A4C, S4C, and IVC views
		Interpretation of LV systolic function, pericardial effusion, and IVC
4	Vascular	Acquisition of lower extremity veins from common femoral vein to popliteal vein
		Compression study of lower extremity veins
5	Lung	Acquisition of views of lung parenchyma and costophrenic recesses
		Recognition of normal lung ultrasound findings (i.e., lung sliding, A-lines)
6	Abdomen	Acquisition of views of kidneys, gallbladder, aorta, bladder
		Recognition of normal findings of kidneys, gallbladder, aorta, bladder

Abbreviations: FOCUS, focused cardiac ultrasound; PLAX, parasternal long-axis view; PSAX, parasternal short-axis view (mid-ventricular level); A4C, apical four-chamber view; S4C, subcostal four-chamber view; IVC, inferior vena cava view; LV, left ventricular.

All course participants completed a pre- and post-course written examination that was administered live at the start and finish of each course. The examinations included 30 multiple-choice questions on fundamentals of ultrasound, FOCUS, lung, lower extremity vascular, and abdominal ultrasound. Each question had an ultrasound video. The first 20 questions tested simple image interpretation (3 points per question), and the remaining 10 questions were case based to test clinical integration (4 points per question). The pre- and post-course examinations were different, but the question objectives were the same. The change in pre- and post-course examination scores was used to evaluate educational effectiveness of the training course. Pre- and post-course examination scores were evaluated by clinical rank, years of POCUS experience, and frequency of POCUS use.

### Participants

Course participants included faculty physicians, residents, and medical students in 5th year and above. We posted emails inviting participants to attend the course on the mailing lists of various academic groups, including the Society of Hospital General Medicine, the Japan Primary Care Association, and freestanding academic groups for hospitalists, primary care physicians, intensivists, emergency physicians, and others. Individuals were invited to participate on a first-come, first-serve basis. No restrictions were placed on years in practice, clinical rank (junior resident, senior resident, or faculty), or departmental affiliation. In Japan, national training regulations require junior residents to complete a 2-year multi-specialty internship with rotations in internal medicine, surgery, emergency medicine, and other specialties after graduation from medical school. During this stage, they are known as junior residents. Afterwards, each resident receives 3 years of training in their specialty of choice, during which they are known as senior residents. After they complete their senior resident training and pass their medical specialty examination, they become an attending physician or specialist. Physicians who had completed their senior resident training were defined as faculty in this study. Medical school is 6 years in Japan. After passing computer-based knowledge testing and an Objective Structured Clinical Examination (OSCE) at the end of the 4th year, medical students participate in clinical clerkships during the 5th and 6th years. Our POCUS training course was open to 5th- and 6th-year medical students.

### Data collection

All course participants completed a pre-course questionnaire about their department, years in practice, clinical rank, years of POCUS experience, and frequency of POCUS use. The pre-course exam was administered at the beginning of each course, and answer sheets were collected immediately. The exam was scored by administrative staff, but the scores and correct answers were not given to the participants. The post-course exam was administered at the end of each course, and afterwards, answer sheets were collected, and correct answers and explanations were provided for each question. Participants were not allowed to take a copy of the examination home.

### Ethical approval and consent

The study was conducted in accordance with the Declaration of Helsinki and approved by the Institutional Review Board of the Tokyo Bay Urayasu Ichikawa Medical Center (protocol code 265, date of approval: July 20th, 2017) and Tokyo Medical and Dental University (protocol code M2019-085, date of approval: August 27th, 2019). Written informed consent was obtained from all participants involved in the study. Data from participants who did not consent to participate in the study or did not complete the training course, course questionnaire, or pre- and post-course exams were excluded.

### Statistical analysis

A paired t-test was used to compare the pre- and post-course examination scores. Student’s t-test was used to analyze the pre- and post-course examination scores and the change in scores by frequency of POCUS use. One-way analysis of variance and Bonferroni correction were used to analyze the scores and changes in scores by years of POCUS experience and clinical rank. Comparisons were also made by years of experience and frequency of use, with groups stratified by clinical rank. Data analysis was performed using STATA 17.0.

## Results

**Figure 2.**
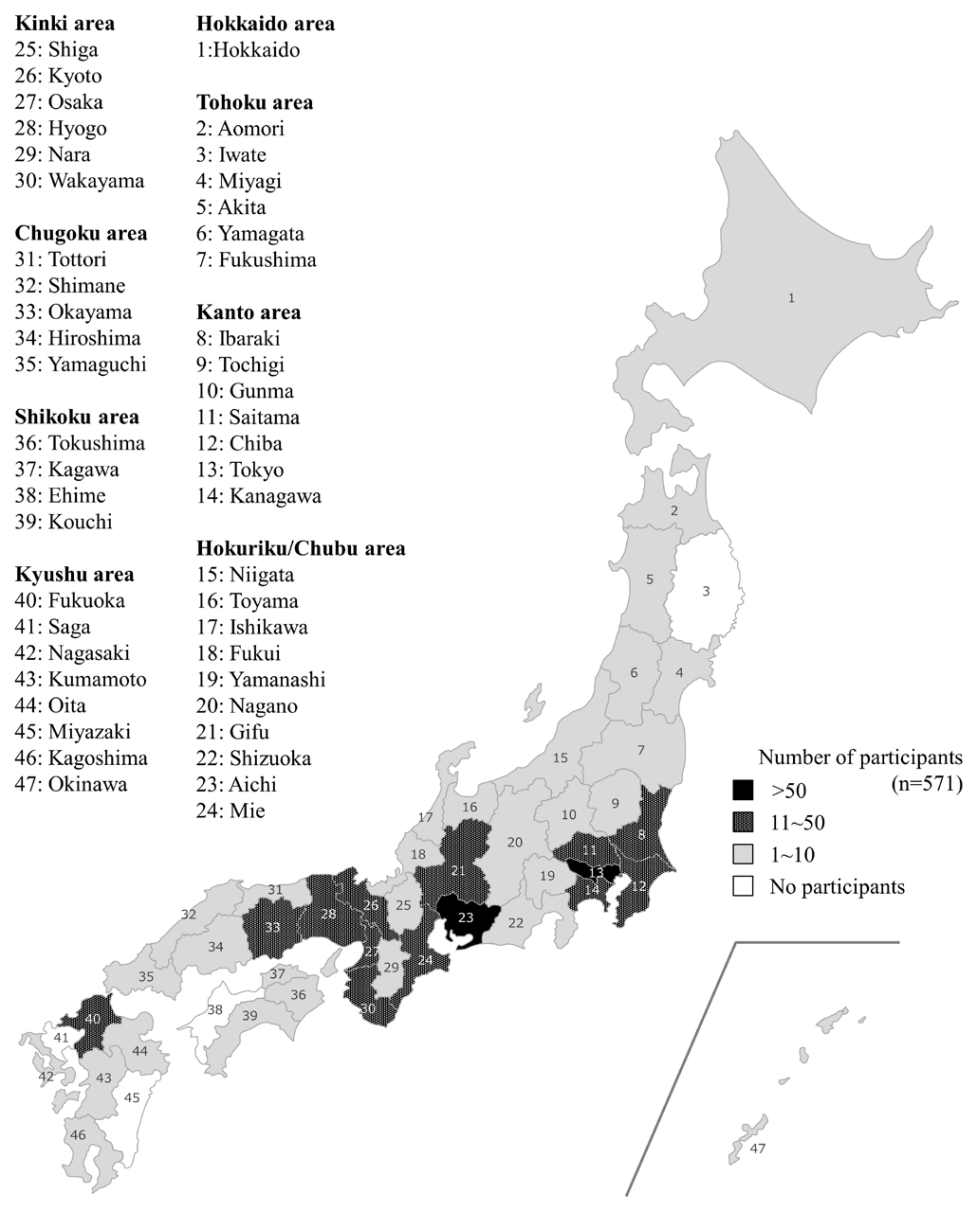
Distribution of course participants by prefecture across Japan.

A total of 630 learners participated in 11 POCUS training courses from 2017 to 2019. No participants had previously attended a POCUS training course. Of the 630 participants, 59 were excluded for not completing both the pre- and post-course examinations (n = 49) or the pre-course questionnaire (n = 10). Data were analyzed from 571 participants in total from across Japan ( Figure 2). Participant characteristics are shown in Table 2.

**Table 2.** Characteristics of course participants (n = 571).

Characteristic	n	(%)
**Gender**		
Male	401	(70)
Female	170	(30)
**Practice setting**		
Private clinic	10	(2)
Community hospital	354	(62)
University hospital	207	(36)
**Clinical rank**		
Medical student *	29	(5)
Junior resident **	281	(49)
Senior resident ***	122	(22)
Attending physician ****	139	(24)
**Specialty**		
IM, subspecialty of IM, GM	186	(33)
Critical care, emergency medicine	46	(8)
Family medicine	8	(1)
Other specialty	21	(4)
Junior resident	281	(49)
Medical student *	29	(5)
**Years of POCUS experience**		
Novice	115	(20)
<2 years	310	(54)
≥2 years	146	(26)
**Frequency of POCUS use**		
*Any ultrasound application*		
Less than once per week	371	(65)
Once or more per week	200	(35)
*Cardiac ultrasound*		
Less than once per week	456	(80)
Once or more per week	115	(20)
*Lung and diaphragm ultrasound*		
Less than once per week	538	(94)
Once or more per week	33	(6)
*Vascular ultrasound of lower* *extremities*		
Less than once per week	554	(97)
Once or more per week	17	(3)
*Abdominal ultrasound*		
Less than once per week	443	(78)
Once or more per week	128	(22)

*Fifth- and sixth-year medical students. **Junior resident: physicians in postgraduate years 1 and 2. ***Senior resident: physicians in postgraduate years 3 to 5 pursuing specialty training after completing junior residency. ****Attending physicians after completion of senior residency. Abbreviations: IM, internal medicine; GM, general medicine; POCUS, point-of-care ultrasound.

### Pre- and post-course examination scores

**Figure 3.**
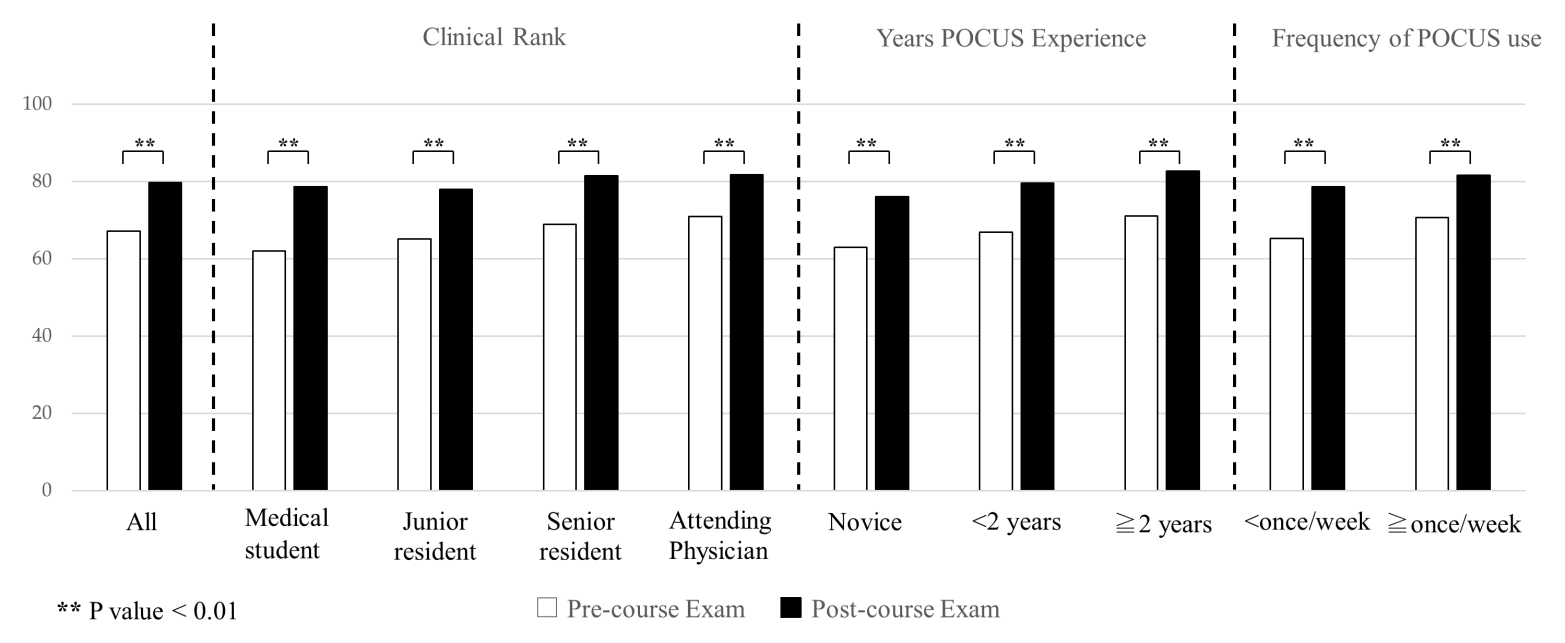
Mean pre- and post-course exam scores by clinical rank, year of POCUS experience, and frequency of POCUS use. Abbreviation: POCUS, point-of-care ultrasound.

The overall mean pre- and post-course examination scores were 67.2 (standard deviation [SD] 12.3) and 79.7 (SD 9.7), respectively. A score breakdown by clinical rank, years of POCUS experience, and frequency of POCUS use is shown in Table 3. The post-course scores were significantly higher than the pre-course scores in all groups (p < 0.01) ( Figure 3).

**Table 3.** Mean pre- and post-course examination scores.

	Mean pre-course exam score (SD)	Mean post-course exam score (SD)
**Overall**	67.2 (12.3)	79.7 (9.7)
**Clinical rank**		
Medical student *	62.0 (13.1)	78.6 (8.5)
Junior resident **	65.1 (11.2)	78.0 (10.3)
Senior resident ***	68.9 (13.0)	81.4 (8.7)
Attending physician ****	70.9 (12.6)	81.7 (8.8)
**Years of ultrasound** **experience**		
Novice	63.0 (11.6)	76.0 (10.4)
<2 years	66.9 (11.2)	79.6 (9.5)
≥2 years	71.1 (13.8)	82.7 (8.5)
**Frequency of** **POCUS use**		
Less than once per week	70.6 (10.9)	81.6 (9.1)
Once or more per week	65.3 (12.6)	78.6 (9.9)

*Fifth- and sixth-year medical students **Junior resident: physicians in postgraduate years 1 and 2 ***Senior resident: physicians in postgraduate years 3 to 5 pursuing specialty training after completing junior residency ****Attending physicians after completion of senior residency Abbreviations: POCUS, point-of-care ultrasound; SD, standard deviation.

### Pre-course examination score analysis

**Figure 4.**
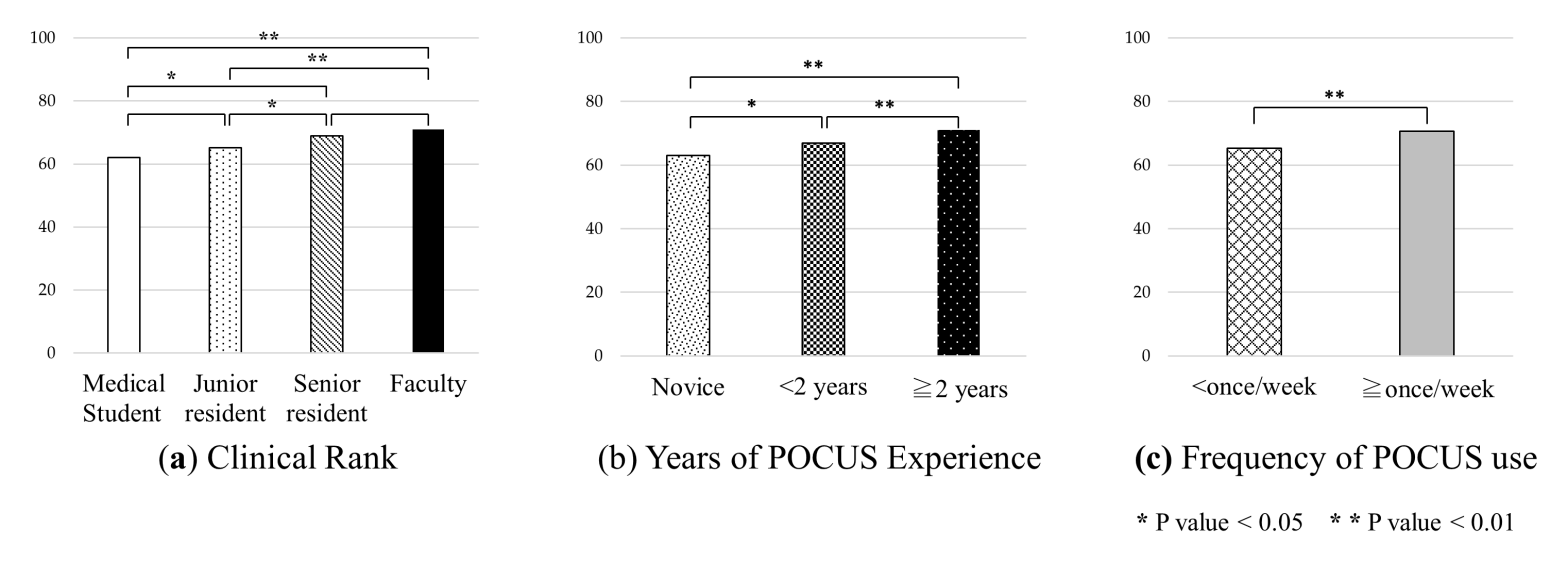
Comparison of pre-course exam scores by clinical rank, years of POCUS experience, and frequency of POCUS use. Abbreviation: POCUS, point-of-care ultrasound.

Pre-course examination scores were compared by clinical rank, years of POCUS experience, and frequency of use ( Figure 4). There were significant differences between students and senior residents (p = 0.03), students and faculty (p < 0.01), junior residents and senior residents (p = 0.03), and junior residents and faculty (p < 0.01). There were also significant differences between all three groups by years of POCUS experience and between two groups by frequency of POCUS use (p < 0.01).

**Figure 5.**
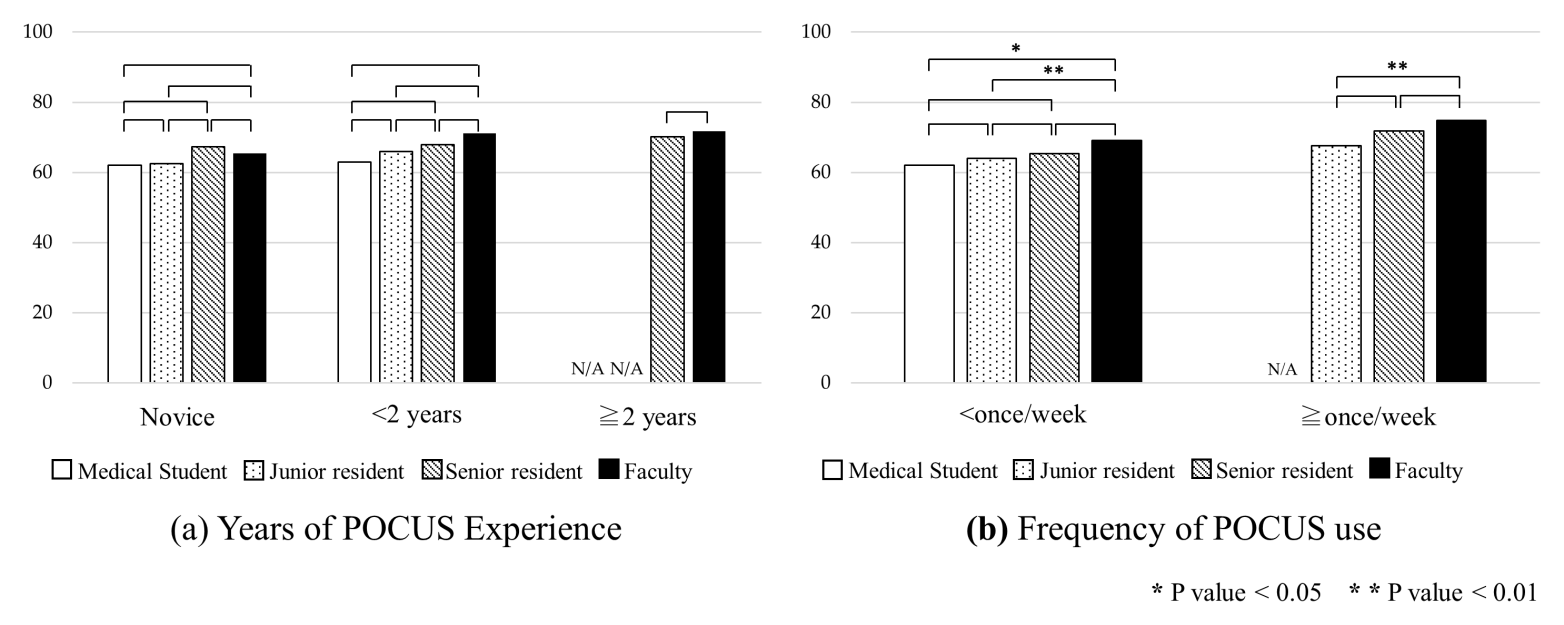
Comparison of pre-course exam scores by years of POCUS experience and frequency of POCUS use, stratified by clinical rank. Abbreviation: POCUS, point-of-care ultrasound.

Next, we compared pre-course scores by years of POCUS experience and frequency of POCUS use, stratified by clinical rank ( Figure 5). In the group with no ultrasound experience (n = 115), the pre-course exam scores were 62.0 (SD 13.3) for medical students, 62.5 (SD 10.9) for junior residents, 67.4 (SD 14.2) for senior residents and 65.5 (SD 10.1) for faculty. There were no significant differences between any of the groups. When comparing groups by frequency of POCUS use, there were significant differences in the group using ultrasound less than once per week (n = 371) between medical students and faculty (p = 0.04) and junior residents and faculty (p < 0.01). In the group using ultrasound at least once per week (n = 200), there was a statistically significant difference between junior residents and faculty (p < 0.01).

### Analysis of differences in pre- and post-course examination scores

**Figure 6.**
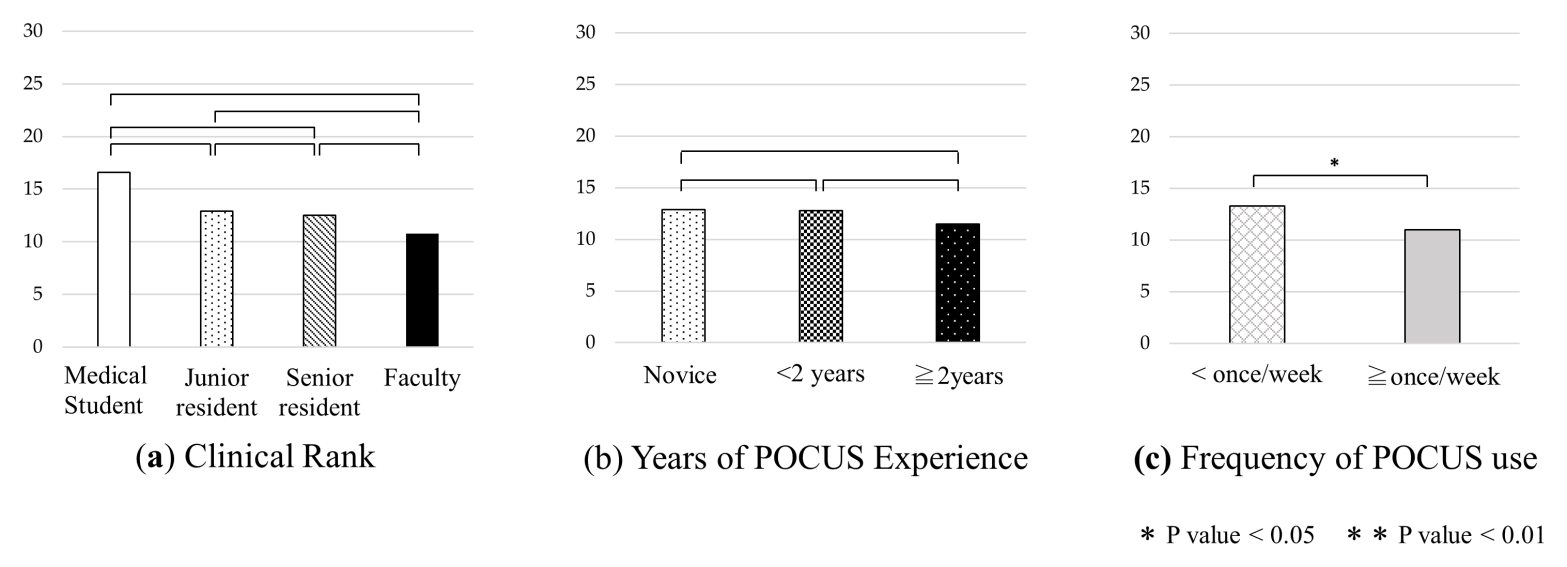
Comparison of change in pre- and post-course exam scores by clinical rank, years of POCUS experience, and frequency of POCUS use. Abbreviation: POCUS, point-of-care ultrasound.

The mean overall difference in pre- and post-course examination scores was 12.5 (SD 11.6) ( Figure 6). Although there was a significant increase from pre- to post-course exam scores in all groups ( Figure 3), there were no statistically significant differences in the change of pre- and post-course exam scores between any of the groups by clinical rank or years of POCUS experience ( Figure 6). The change in exam scores for those using ultrasound less than once a week versus more than once a week were 13.3 (SD 12.3) and 11.0 (SD 10.1) which was statistically significant (p = 0.03).

**Figure 7.**
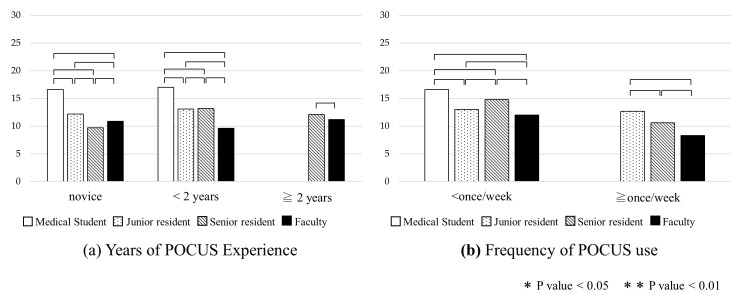
Comparison of change in pre- and post-course exam scores by years of POCUS experience and frequency of POCUS use, stratified by clinical rank. Abbreviation: POCUS, point-of-care ultrasound.

Additional analyses were conducted comparing change in pre- and post-course exam scores by years of POCUS experience and frequency of POCUS use, stratified by clinical rank. These analyses did not find any statistically significant differences between any of the groups ( Figure 7).

## Discussion

We investigated the educational effectiveness of a standardized 2-day hands-on POCUS training course developed for physicians across Japan and the change in pre- and post-course exam scores in relationship to participants’ clinical rank, years of POCUS experience, and frequency of POCUS use in clinical practice. Data were collected from a total of 571 physicians across 11 courses from 2017 to 2019, the largest collection of data from a POCUS training course to date. There were three key findings of our study. First, the course was effective in educating participants regardless of baseline differences, including clinical rank, years of POCUS experience, and frequency of POCUS use. Second, pre-course exam scores differed significantly by clinical rank, ultrasound experience, and frequency of use, but the improvement in post-course exam scores did not differ significantly, signifying the training course was beneficial to give all learners a similar level of POCUS knowledge post-course, regardless of their baseline POCUS knowledge. Third, this course was shown to be effective and could serve as a possible model to standardize POCUS training for a broad range of learners.

Lack of training and training-related barriers, including availability, funding, and time for training, have become the greatest barriers to POCUS implementation in healthcare systems ( Eisen *et al.*, 2010; Nathanson *et al.*, 2023; Resop *et al.*, 2023; Soni *et al.*, 2016; Stowell *et al.*, 2018; Williams *et al.*, 2022; Wong *et al.*, 2020). Eventually, POCUS training will become a standard curricular component in undergraduate and graduate medical education, bridging the training gap that is prevalent today. However, currently, most clinicians receive POCUS training through immersive, hands-on courses. Such courses have been developed as continuing medical education courses for physicians in-practice, and as ‘boot camps’ for medical students and residents that are typically 1 to 3 days in duration. These courses require live, hands-on scanning practice which is costly and resource-intensive, precluding many institutions from being able to offer POCUS training. We have demonstrated that learners from a wide range of clinical backgrounds can achieve a similar level of POCUS knowledge after participating in the same POCUS training course. Thus, institutions seeking to implement basic POCUS use across clinical specialties may be able to streamline training efforts by having clinicians participate in the same standardized basic POCUS training course, resulting in all participants having a similar level of POCUS knowledge upon completion.

POCUS findings are integrated with other clinical findings to make clinical decisions, and therefore, both clinical experience and ultrasound skills are important. Generally, it is well accepted that higher clinical rank signifies greater clinical experience. However, ultrasound skills cannot be presumed to be better with higher clinical rank, and on the contrary, trainees are more likely to have been exposed to ultrasound during medical school or residency training and be better skilled in POCUS. In our study, faculty with more years of POCUS experience and who used POCUS frequently had higher pre-course scores. However, there was no difference in pre-course exam scores by clinical rank for participants with similar levels of ultrasound experience. These data suggest that years of POCUS experience may have more influence on POCUS skills than clinical rank.

This study has limitations. Competency in POCUS requires mastery of basic ultrasound knowledge and skills in image acquisition, image interpretation, and clinical integration. Our pre-/post-course examination assessed basic ultrasound knowledge, image interpretation, and clinical integration skills, but did not assess image acquisition skills. Additionally, we were not able to assess knowledge or skills retention post-course. A strength of our study is the large number of participants (n = 571) from whom we collected data which is greater than other similar studies.

Future studies shall track course participants longitudinally to assess POCUS skills and knowledge retention several months post-course, as well as evaluate incorporation of POCUS use into clinical practice. Elucidating barriers and facilitators to POCUS use in clinical practice will improve our understanding of how to systematically implement and standardize POCUS use across healthcare systems. Finally, a basic POCUS skill certification process is not currently in place in Japan, and developing such a certification will help maintain standards and promote safe POCUS use in different clinical settings.

## Conclusion

A standardized, hands-on POCUS training course significantly improved POCUS knowledge among all participants. Although pre-course exam scores differed by clinical rank, years of POCUS experience, and frequency of POCUS use, these factors did not impact the course’s educational effectiveness based on post-course exam scores. Upon completion of the POCUS training course, all participants had a similar level of POCUS knowledge despite baseline differences. Future studies shall evaluate POCUS skills and knowledge retention and incorporation of POCUS use into clinical practice several months post-course.

## Data Availability

Zenodo: Development of a national point-of-care ultrasound training course for physicians in Japan: A 3-year evaluation. https://doi.org/10.5281/zenodo.7947863 ( Yamada *et al*., 2023). This project contains the following underlying data: POCUS raw data revised 2.xls Codebook 0518.xlsx (coding keys and pre-course questionnaire items) Data are available under the terms of the Creative Commons Attribution 4.0 International license (CC-BY 4.0). Responses to each pre- and post-course exam question cannot be shared publicly because the results were recorded on paper media and transcribing them into digital format in a sharable format at this point is not feasible. The “POCUS raw data revised 2.xls” file contains individual level scored data. All the raw data (before undergoing scoring) can be made available by the authors to researchers/reviewers, upon reasonable request. Please contact the corresponding author Taro Minami to request access. The additional data that cannot be shared includes the study participants’ personal identifiers. As required by ethical guidelines and privacy laws, these data have been securely stored and protected to uphold the participants’ confidentiality rights. Sharing this data type would violate our commitment to participants and potentially infringe on legal obligations. The exam questions cannot be disclosed because the course is still being held as an educational course, and the questions should be protected. The questions will be made available by the authors to researchers/reviewers, upon reasonable request. Please contact the corresponding author Taro Minami to request access.

## References

[ref-1] American College of Physicians: Point of care ultrasound (POCUS) pathway for internal medicine.2023; [accessed 2023 Jan 13]. Reference Source

[ref-2] Eisen LA Leung S Gallagher AE : Barriers to ultrasound training in critical care medicine fellowships: a survey of program directors. *Crit Care Med.* 2010;38(10):1978–1983. 10.1097/CCM.0b013e3181eeda53 20657275

[ref-3] Greenstein YY Littauer R Narasimhan M : Effectiveness of a critical care ultrasonography course. *Chest.* 2017;151(1):34–40. 10.1016/j.chest.2016.08.1465 27645689

[ref-4] Nathanson R Williams JP Gupta N : Current use and barriers to point-of-care ultrasound in primary care: a national survey of VA medical centers. *Am J Med.* 2023;136(6):592–595. e2, S0002-9343(23): 00108-0. 10.1016/j.amjmed.2023.01.038 36828205

[ref-5] Resop DM Basrai Z Boyd JS : Current use, training, and barriers in point-of-care ultrasound in emergency departments in 2020: a national survey of VA hospitals. *Am J Emerg Med.* 2023;63:142–146. 10.1016/j.ajem.2022.09.019 36182580

[ref-7] Singh J Matern LH Bittner EA : Characteristics of simulation-based point-of-care ultrasound education: a systematic review of MedEdPORTAL curricula. *Cureus.* 2022:14(2): e22249. 10.7759/cureus.22249 35186609 PMC8849358

[ref-6] Society of Hospital Medicine: POCUS certification of completion.2023; [accessed 2023 Jan 13]. Reference Source

[ref-8] Soni NJ Arntfield R Kory P : Point-of-care ultrasound.2nd ed. Philadelphia: Elsevier.2020. Reference Source

[ref-9] Soni NJ Reyes LF Keyt H : Use of ultrasound guidance for central venous catheterization: a national survey of intensivists and hospitalists. *J Crit Care.* 2016;36:277–283. 10.1016/j.jcrc.2016.07.014 27491563

[ref-10] Soni NJ Schnobrich D Mathews BK : Point-of-care ultrasound for hospitalists: a position statement of the Society of Hospital Medicine. *J Hosp Med.* 2019;14:E1–E6. 10.12788/jhm.3079 30604779 PMC8021128

[ref-11] Stowell JR Kessler R Lewiss RE : Critical care ultrasound: a national survey across specialties. *J Clin Ultrasound.* 2018;46(3):167–177. 10.1002/jcu.22559 29131347

[ref-16] Williams JP Nathanson R LoPresti CM : Current use, training, and barriers in point-of-care ultrasound in hospital medicine: a national survey of VA hospitals. *J Hosp Med.* 2022;17(8):601–608. 10.1002/jhm.12911 35844080

[ref-12] Wong J Montague S Wallace P : Barriers to learning and using point-of-care ultrasound: a survey of practicing internists in six North American institutions. *Ultrasound J.* 2020;12(1): 19. 10.1186/s13089-020-00167-6 32307598 PMC7167384

[ref-13] Yamada T Minami T Kitano Y : Development of a national point-of-care ultrasound training course for physicians in Japan: A 3-year evaluation [Data set]. *Zenodo* .2023. 10.5281/zenodo.7947863 PMC10831232 38303735

[ref-14] Yamada T : Dai ikkai point-of-care tyouonpa kosyukai [First point-of-care ultrasound workshop]. *Nihon Naikagakkai Zasshi.* 2020;109(7):1448–1452. Japanese.

[ref-15] Yamada T Minami T Soni NJ : Skills acquisition for novice learners after a point-of-care ultrasound course: does clinical rank matter? *BMC Med Educ.* 2018;18(1): 202. 10.1186/s12909-018-1310-3 30134975 PMC6106885

