# Supplementary material for: Development of a national point-of-care ultrasound training course for physicians in Japan: A 3-year evaluation
Source: MedEdPublish (2016). 2023 Oct 25;13:223. [Version 1] doi: 10.12688/mep.19679.1 (PMC10831232; doi:10.12688/mep.19679.1)
Supplement: Peer review history

# Reviewer report: Reviewer response for version 1

**DOI:** 10.21956/mep.21080.r35351

**Published:** 2024-01-31

**Authors:** John Stephens

**Affiliations:** 1 University of Sunderland, Sunderland, UK

Thank you for the invitation to review the article. Overall the work is contemporary; an enjoyable and informative article that has great potential to direct further research of the subject area for a wider audience as well as the research team. This is articulated by the team at page 9, paragraph 5. As well as the proposed longitudinal work, research involving qualitative methodologies could, for example, explore the learning processes and application to practice for different professions, and differing levels of experience.

 The abstract accurately conveys the nature of the research providing a useful summary for anyone sifting evidence as part of literature search of the subject area. The introduction and literature although very succinct does provide a clear rationale for the work undertaken. 

 Methods are reported in a transparent fashion, with a wide range of participants which I feel adds to interest for the research. Statistical analysis is succinctly and accurately reported. This section would have possibly been enhanced further through inclusion of which members of the team had carried out the analysis.

 Results are fairly comprehensive and clearly presented with very good use of tabulation and charts. Although a quite 'busy' section the accompanying text provides a valuable report to inform a balanced discussion and an appropriate conclusion.

Have any limitations of the research been acknowledged?

Yes

Is the work clearly and accurately presented and does it cite the current literature?

Yes

If applicable, is the statistical analysis and its interpretation appropriate?

Yes

Are all the source data underlying the results available to ensure full reproducibility?

Yes

Is the study design appropriate and is the work technically sound?

Yes

Are the conclusions drawn adequately supported by the results?

Yes

Are sufficient details of methods and analysis provided to allow replication by others?

Yes

Reviewer Expertise:

education, healthcare professional education, curriculum design, cardiorespiratory physiotherapy.

I confirm that I have read this submission and believe that I have an appropriate level of expertise to confirm that it is of an acceptable scientific standard.

# Reviewer report: Reviewer response for version 1

**DOI:** 10.21956/mep.21080.r35359

**Published:** 2024-01-31

**Authors:** Ajay Bhasin

**Affiliations:** 1 Northwestern University Feinberg School of Medicine, IL, USA

Well written article describing the utility and effectiveness of a national point of care ultrasound training course for physicians in Japan. It describes nicely the methods used to train learners, differences between baseline capabilities and, interestingly, convergence of post-training capabilities to nearly the same level of POCUS knowledge. 

 Importantly, though the study is well conducted, a crucial element not evaluated is the ability of learners to acquire images themselves, which is a key feature of POCUS. As this cannot be reported in the current study, future studies should evaluate similarly the baseline skill and image acquisition ability of various learners though a national standardized training course. 

 What would be interesting to report is the type of question missed / gotten wrong on the assessments by level of training and/or rank and how they changed post-training.

Have any limitations of the research been acknowledged?

Yes

Is the work clearly and accurately presented and does it cite the current literature?

Yes

If applicable, is the statistical analysis and its interpretation appropriate?

Yes

Are all the source data underlying the results available to ensure full reproducibility?

Yes

Is the study design appropriate and is the work technically sound?

Yes

Are the conclusions drawn adequately supported by the results?

Yes

Are sufficient details of methods and analysis provided to allow replication by others?

Yes

Reviewer Expertise:

Point of care ultrasound, data science

I confirm that I have read this submission and believe that I have an appropriate level of expertise to confirm that it is of an acceptable scientific standard.

# Reviewer report: Reviewer response for version 1

**DOI:** 10.21956/mep.21080.r35352

**Published:** 2023-11-27

**Authors:** Michael Janjigian

**Affiliations:** 1 New York University Grossman School of Medicine, New York, USA

Overall this is an excellent manuscript which contributes to our understanding of POCUS training programs. I have a few minor points of clarification and to correct what seems like an error in Table 3. 

 The introduction is clearly written with a clearly defined study objective.

 Consider adding a cost analysis of the course given your recommendation to expand this course nationally to resource limited areas. 

 Given that the results of this study revolve heavily around self-reported years of POCUS experience (Novice, <2 year, >2 year) and frequency of use, I would want more background on how these questions were framed in the survey or perhaps an explanation of why participants of an introductory course would have prior experience or use POCUS routinely. 

 For frequency of POCUS use, is it possible participants reported ultrasound guided venipuncture or IV access or other common procedures?

 Do students or residents receive any formal or informal POCUS training apart from this course?

 The bar graph in Figure 3 shows the pre-test scores for frequency of use reversed compared to Table 3, there appears to be an error. Figure 4 matches Figure 3 so I suspect the error is in Table 3.

 The discussion includes medical students as physicians which would not be defined this way to my understanding. Consider changing the language accordingly.

 “Second, pre-course exam scores differed significantly by clinical rank, ultrasound experience, and frequency of use, but the improvement in post-course exam scores did not differ significantly, signifying the training course was beneficial to give all learners a similar level of POCUS knowledge post-course, regardless of their baseline POCUS knowledge.” 

- Did you test the difference in post-test outcomes across groups or just the change from pre- to post- (absolute or the change)? If the latter, which is how I read your report, then this statement should be reworded to focus on the change in knowledge, not the final level. Or report the statistics on the final test score across groups which visually appears to be similar.

As mentioned above regarding resources required to run the program, paragraph 2 of the discussion comments that resources are required to run a 2-day course but does not explain how expanding this course broadly (students, residents, faculty, across the country) would address the resource problem apart from providing a standardized curriculum.

 An additional limitation is that you are using a test as a surrogate for POCUS competency rather than a hands-on test or OSCE. Clinical integration is incompletely assessed through a test question. Though I agree that a test is a reasonable surrogate, it would be worth commenting on limitations in assessments. Another limitation is that much of the quantitative data is from self-reported experience and frequency of use, which is not well characterized in the manuscript and fundamentally qualitative in nature. 

 In the conclusion, you state that “all participants” improved their knowledge. You have shown that the cohort as a whole improved knowledge rather than each individual.

Have any limitations of the research been acknowledged?

Yes

Is the work clearly and accurately presented and does it cite the current literature?

Yes

If applicable, is the statistical analysis and its interpretation appropriate?

Yes

Are all the source data underlying the results available to ensure full reproducibility?

Yes

Is the study design appropriate and is the work technically sound?

Yes

Are the conclusions drawn adequately supported by the results?

Yes

Are sufficient details of methods and analysis provided to allow replication by others?

Yes

Reviewer Expertise:

Point-of-care ultrasound programmatic development and assessment

I confirm that I have read this submission and believe that I have an appropriate level of expertise to confirm that it is of an acceptable scientific standard.
